# 

**Published:** 1890-01

**Authors:** 


### Books and Pamphlets Received.

"Oxygen and other Gases in Medicine and Surgery." Di-Marquay, Trans. Williams. Published by A. F. Davis.

Shoemaker & Auldi—"Materia Medica, Pharmacology, etc." Vol. I. Published by F. A. Davis.

"The Physician Himself." Revised Edition. Published by F. A. Davis.

"The Essentials of Materia Medica and Therapeutics." By Henry Morris.

"Saunder's Question Compends."

"Physician's Pocket Reference Book and Visiting List, 1890." J. H. Chambers & Co., St. Louis.

